# Protective Effect of Xiao-Xu-Ming Decoction-Mediated Inhibition of ROS/NLRP3 Axis on Lipopolysaccharide-Induced Acute Lung Injury *In Vitro* and *In Vivo*

**DOI:** 10.1155/2021/8257495

**Published:** 2021-09-27

**Authors:** Yijin Xiang, Min Cai, Xiangting Li, Xuxia Bao, Dingfang Cai

**Affiliations:** Department of Integrative Medicine, Zhongshan Hospital, Fudan University, Institutes of Integrative Medicine, Fudan University, Development Project of Shanghai Peak Disciplines-Integrative Medicine, Shanghai 200032, China

## Abstract

**Background:**

As a traditional Chinese medicine prescription, Xiao-Xu-Ming decoction (XXMD) could reduce the incidence of lung infection of patients with cerebral infarction. Nonetheless, the therapeutic mechanisms of XXMD in acute lung injury (ALI) remain to be elucidated. Our study was aimed to assess the effects of XXMD protects against ALI.

**Methods:**

ALI model was induced by intraperitoneal injection of lipopolysaccharide (LPS) *in vivo*. *In vitro*, human pulmonary alveolar epithelial cells (HPAEpiC) were treated with XXMD and were followed by LPS treatment. The levels of ZO-1, CLDN4, NLRP3, and caspase 1 were detected by Western blot, and the content of IL-1 and IL-18 was determined by ELISA. Transepithelial electrical resistance was used to detect the cell permeability. The reactive oxygen species (ROS) levels within the cells were evaluated by flow cytometry.

**Results:**

Our results showed that XXMD attenuated LPS-induced oxidative stress, barrier dysfunction, and the activation of NLRP3 inflammasome *in vitro*, as evidenced by enhanced ROS production, TEER levels, expression of NLRP3 and caspase 1 (p20) and release of IL-1*β* and IL-18, and weakened cell permeability. In addition, XXMD could counteract the effects of NLRP3 overexpression on HPAEpiC and vice versa. XXMD treatment also ameliorated the degree of neutrophil infiltration, barrier dysfunction, and the activation of NLRP3 in LPS-induced ALI lung tissues *in vivo*.

**Conclusion:**

The findings showed that XXMD could alleviate LPS-induced ALI injury and inhibit inflammation and suppress ROS/NLRP3 signaling pathway, which were involved in these protective effects.

## 1. Background

ALI is a typical lung inflammation, which is a critical symptom that causes edema, hypoxemia, and respiratory distress due to pathogenic factors in the lung tissues [1]. The patient's blood-oxygen binding capacity is ameliorated, which could lead to acute respiratory distress syndrome (ARDS) [2]. ALI develops rapidly and usually coexists with multiple organ dysfunction syndromes, and it is a disease with high morbidity and mortality. Statistical analysis conformed that the fatality rate of ALI/ARDS is still as high as 40% [3]. Endogenous and exogenous injury factors performed a role in the lungs, and they may cause lung cells to be exposed to excessive amounts of reactive oxygen species (ROS), inflammatory cell infiltration, and proinflammatory mediator production, which were also involved in the process of ALI [4]. Clinically, supportive treatment mainly includes small tidal volume ventilation, lung fluid management, and antibacterial therapy. Drug therapy mainly consists of vasodilator inhalation therapy, antioxidant therapy, and ketoconazole therapy. However, in recent years, supportive techniques of treating ALI have improved to a great extent, but, so far, there is no effective treatment. Therefore, exploring an effective treatment strategy for ALI has become a hot spot in clinical research.

Currently, multiple evidences further indicate that many traditional Chinese medicines have been used to treat ALI [5, 6]. As a traditional Chinese medicine formula, Xiao-Xu-Ming decoction (XXMD) is composed of *Ephedrae Herba*, *Cmnamomi Mmulus*, *Chuanxiong Rhizoma*, *Red Ginseng*, *Aconiti Lateralis Radix Praeparata*, *Paeoniae Radix Alba*, *Armeniacae Semen Amarum*, *Zingiberis Rhizoma Recens*, *Scutellariae Radix*, *Radix Stephaniae Thtrandrae,* and *Saposhnikoviae Radix*. XXMD performed predominant protective effects on stroke and other neurological diseases for thousands of years [7, 8]. Interestingly, our previous study demonstrated that XXMD could ameliorate the incidence of lung infection of patients with cerebral infarction. However, the therapeutic mechanisms of XXMD in ALI remain unclear.

Recently, some reports have been used to study the specific descriptions of its mechanisms related to ALI development. It found that the level of ROS was associated with the elevated inflammatory, and oxidative stress produces a large number of ROS in the body, which could cause airway and blood vessel remodeling, invades the lung interstitium to cause pulmonary edema, and causes oxidative damage to lung parenchymal cells [9]. Therefore, oxidative stress-induced damage was thought to be crucial role in the injury of ALI. Report showed that lipopolysaccharide (LPS) activated NLRP3 (NOD-like receptor family, pyrin domain-containing 3) in mouse lung vascular endothelial cells through Toll-Like Receptor 4 (TLR4), and subsequently induced caspase 1 activation in lung endothelial cells [10]. NLRP3 is a danger signal receptor in innate immune cells, which can be activated by a variety of factors, such as pathogens or damage-related molecular patterns [11], and it performs a crucial role in identifying risk factors for innate immunity of the lung injury induced by cardiopulmonary bypass [12]. However, the effects of XXMD on ALI and whether XXMD mediated ROS/NLRP3 pathway to perform the protective effects on ALI remain unclear. Therefore, our study was aimed to investigate the effects and explore the molecular mechanism by which XXMD regulates ROS/NLRP3 inflammasome in ALI induced by LPS.

## 2. Materials and Methods

### 2.1. Reagent and Antibody

HPAEpiC were obtained from ScienCell Research Laboratories (San Diego, CA, USA); Dulbecco's modified Eagle's medium (DMEM; GE Healthcare Life Sciences, Logan, UT); Lipofectamine 2000 (Invitrogen, USA); 1% penicillin-streptomycin mixture (Gibco, Grand Island, NY); N-Acetyl-L-cysteine (NAC; Selleck, Houston, TX, USA); dexamethasone (DEX; Sigma-Aldrich, St. Louis, MO, USA); Cell Counting Kit-8 (CCK-8; Signaling Antibody, College Park, MD, USA); fluorescent 2,7-dichlorodi-hydrofluorescein diacetate (DCFH-DA) probe (Beyotime, China); IL-1*β* and IL-18 ELISA kits (Nanjing Jiancheng Bioengineering Institute); TRIzol (Thermo Fisher Scientific,, USA); NLRP3 (Abcam, Cambridge, MA, USA; ab263899); Zonula Occludens Protein 1 (ZO-1; Abcam; ab96587); Claudin 4 (CLDN4; Proteintech; 16195-1-AP); Caspase-1 (Abcam; ab207802); GAPDH (Proteintech; 60004-1-1G); anti-Horseradish peroxidase- (HRP-) conjugated secondary antibody (Beyotime Biotechnology; A0208 and A0216).

### 2.2. XXMD Preparation

XXMD consists of twelve Chinese herbs, including Radix Paeoniae Alba (9 g), Rhizoma Zingiberis Recens (9 g), Semen Armeniacae Amarum (9 g), Radix Stephaniae Tetrandrae (6 g), Radix Saposhnikoviae (6 g), Radix Scutellariae (6 g), Herba Ephedrae (3 g), Radix Ginseng (3 g), Radix Glycyrrhizae (3 g), Radix Aconiti Lateralis Preparata (3 g), Ramulus Cinnamomi (3 g), and Rhizoma Ligustici Chuanxiong (3 g). All crude herbs were purchased from the Traditional Chinese Medicine Pharmacy of Zhongshan Hospital, Fudan University. XXMD was prepared as previously reported [13].

### 2.3. Cell Culture and Treatment

HPAEpiC (human pulmonary alveolar epithelial cells) were treated in DMEM containing 10% fetal bovine serum and 1% penicillin-streptomycin mixture at 37°C under a humidified atmosphere with 5% CO_2_.

Treatments were divided into five groups. In Group 1, HPAEpiC were treated with XXMD (0.05, 0.1, 0.2, 0.5, 1, and 2 mg/mL) for 3 h and then had LPS (10 mg/L) treatment for 6, 12, and 24 h. In Group 2, the cells were treated with XXMD (0.2, 0.5, and 1 mg/mL) for 3 h and then had LPS (10 mg/L) treatment for 24 h. In Group 3, HPAEpiC were transduced with pLVX-Puro-NLRP3 and blank vector, followed by XXMD (1 mg/mL) treatment for 24 h. In Group 4, HPAEpiC transduced with pLVX-Puro-NLRP3 or blank pLVX-Puro vector were treated with XXMD (1 mg/mL) for 3 h and then treated with LPS (10 mg/L) for 24 h. In Group 5, HPAEpiC were treated with XXMD (1 mg/mL) or ROS inhibitor NAC (1 mM) for 3 h and then with LPS (10 mg/L) for 24 h.

### 2.4. Gene Overexpression

To obtain the NLRP3 overexpression, coding DNA sequence of the genes was successfully cloned in the pLVX-Puro plasmid (Clontech). In order to obtain virus particles, the overexpression level of plasmids was cotransfected with pMD2G and psPAX2 plasmids into 293 T cells. Lipofectamine 2000 (Invitrogen, USA) was used for transfection. The virus particles were obtained 48 h after transfection. Blank pLVX-Puro plasmid (vector) was used as negative control for overexpression experiments.

### 2.5. CCK-8 Assay Detecting Cell Viability

The cells with a number of 3 × 10^3^ per well were performed on 96-well plate and kept in a continuous cell culture overnight. At 0, 6, 12, and 24 h after treatment, each well was added with Cell Counting Kit-8 solution at a volume of 10 *μ*L according to the manufacturer's protocols. After continuing incubation for 1 h, the absorption value of each well was read on a microplate reader at OD 450 nm, and this reading value was used to calculate relative cell viability.

### 2.6. Detection of ROS

A fluorescent DCFH-DA probe was used to evaluate ROS levels within the HPAEpiC cells. Briefly, the DCFH-DA probe was subjected into the cell culture medium to a final concentration of 10 *μ*M. After a 20 min incubation in darkness at 4°C, the fluorescence was determined at an excitation/emission of 485/530 nm using flow cytometry (BD Biosciences, USA).

### 2.7. Cell Permeability Detection

Cell permeability was detected by transepithelial electrical resistance (TEER) FITCDextran transepithelial flux. HPAEpiC were inoculated on top of the pipetting insert in the 24-well pipetting chamber. Then, TEER was detected by Millicell ERS-2, fluorescein isothiocyanate- (FITC-) dextran (1 mg/mL) was added to the dealt cells for 1 h, and the fluorescence intensity of FITC was detected by a microplate reader, using the final detection value of each group minus the basic value as the fluorescence intensity value of FITCDextran to calculate the fluorescence intensity.

### 2.8. IL-1 and IL-18 Were Detected by ELISA

The content of IL-1*β* and IL-18 in HPAEpiC cell supernatant and bronchoalveolar lavage fluid was analyzed using enzyme-linked immunosorbent assay (ELISA) kits followed by the manufacturer's instructions, respectively.

### 2.9. Quantitative Real-Time PCR Analysis

Total RNA was made from HPAEpiC using TRIzol (Thermo Fisher Scientific, USA) in two steps. Firstly, RNA was subjected to reverse transcription into cDNA by the reaction system of the PrimeScript kit that provided by Takara Biotechnology. Secondly, the PCR amplification of cDNA product was subjected to the reaction system of the SYBR green PCR master mix provided by Applied Biosystems. The applied primer sequences are listed as follows: NLRP3-F: 5ʹ-GAGCCTCAACAAACGCTAC-3ʹ; NLRP3-R: 5ʹ-GACGCCCAGTCCAACATC-3ʹ; GAPDH-F: 5ʹ-AATCCCATCACCATCTTC-3ʹ; and GAPDH-R: 5ʹ-AGGCTGTTGTCATACTTC-3ʹ. The level of GAPDH was the internal control.

### 2.10. Western Blot Analysis

The protein of each sample was separated from HPAEpiC and lung tissues and then was added with a mixture of protease inhibitors and loaded on a sodium dodecyl sulfate-polyacrylamide gel electrophoresis gel, which was subjected to electrophoresis for protein separation. Then, the gel proteins were transferred onto a nitrocellulose membrane and then incubated with primary antibodies anti-NLRP3, anti-ZO-1, anti-CLDN4, anti-caspase 1, GAPDH, and anti-HRP-conjugated secondary antibody. Finally, the membrane was soaked by enhanced chemiluminescence system from Bio-Rad Laboratories to visualize specific signals and photographs.

### 2.11. Animal Experiments

Male C57BL/6J mice of 6–8 weeks were obtained from Jackson Laboratory (Bar Harbor, ME) and randomly designed into the following groups (*n* = 6): control group, LPS group, LPS + XXMD group, LPS + NAC, and LPS + DEX group. ALI model was induced by LPS (intraperitoneal injection, 10 mg/kg) with or without intragastric administration of XXMD (50 and 100 mg/kg; twice a day), NAC (400 mg/kg; once a day), or DEX (2.5 mg/kg; once a day) 3 days after LPS treatment. After 24 h of XXMD, NAC, or DEX administration, mice were anesthetized intraperitoneally with urethane (1.25 g/kg, Sigma-Aldrich, St. Louis, MO, USA), killed by exsanguinations, and lung was collected for hematoxylin-eosin (H&E) and immunohistochemistry (IHC) staining as previously described [8]. All experiments were approved by the Ethics Committee on Animal Use from the Zhongshan Hospital, Fudan University. All research involving animals was conducted according to the Guide for the Care and Use of Laboratory Animals (8th edition, National Academies Press).

### 2.12. Statistical Analysis

The data statistics and analysis were performed by GraphPad Prism 8.0.2. A comparison between two groups used Student's *t*-test, and more than three groups used analysis of variance (ANOVA). The data were expressed as mean ± standard deviation (SD) and *P* < 0.05 was identified as significant difference.

## 3. Results

XXMD inhibits oxidative stress, NLRP3 inflammasome activation, and barrier dysfunction in HPAEpiC induced by LPS.

**Figure 1:**
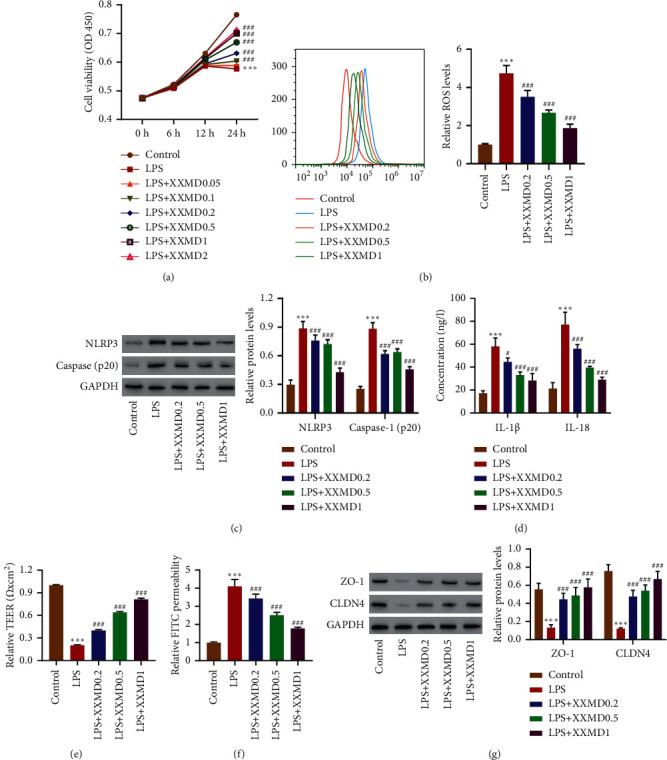
Effect of XXMD on ROS production, hyperpermeability, and NLRP3 inflammasome in HPAEpiC after LPS treatment. (a) HPAEpiC were treated with XXMD (0.05, 0.1, 0.2, 0.5, 1, and 2 mg/mL) for 3 h and then treated with LPS (10 mg/L) for 6, 12, and 24 h, and the cell viability was assessed. HPAEpiC were treated with XXMD (0.2, 0.5, and 1 mg/mL) for 3 h and treated with LPS (10 mg/L) for 24 h. The (b) ROS production, (c) the TEER analysis, and (d) FITC permeability, (e, f) the level of ZO-1, CLDN4, NLRP3, and caspase 1, and (g) the levels of IL-1*β* and IL-18 were determined. Data are presented as the means ± SD, *n* = 3. ^*∗∗∗*^*P* < 0.001 compared with control; ^#^*P* < 0.05, ^###^*P* < 0.001 compared with LPS.

To investigate the mechanisms of XXMD on ALI progression, we mimicked the *in vivo* ALI model using LPS-induced HPAEpiC, and then cell viability was measured. It demonstrated that the viability of HPAEpiC induced by LPS were significantly reduced; however, XXMD could significantly increase the cell viability (Figure 1(a)). ROS as an important secondary messenger participates in various signal cascades and increases the degree of inflammation and tissue damage; oxidative stress was involved in the process of ALI [14]. Compared with the control group, LPS significantly enhanced the level of ROS (*P* < 0.001); however, XXMD significantly decreased this production (*P* < 0.001) in a dose-dependent way (Figure 1(b)).

It was reported that excessive ROS could activate NLRP3 inflammasome and then lead to the activation of caspase 1 and the release of IL-1*β* and IL-18. The results of Western blot and ELISA showed that LPS treatment could significantly increase the levels of NLRP3, caspase 1 (p20), IL-1*β*, and IL-18 as compared with the control group; however, these levels were markedly reversed by XXMD treatment as compared with LPS group (Figures 1(c) and 1(d)).

In the development of ALI, the NLRP3 activation was an important factor in inflammation and then led to the conduction of a series of signal pathways, which ultimately leads to changes in permeability [15]. Our results indicated that LPS could decrease the relative value of TEER (*P* < 0.001). However, XXMD could significantly increase this trend (*P* < 0.001), compared with LPS group (Figure 1(e)). In addition, LPS increased the cell permeability, compared with control group; meanwhile, XXMD significantly reduced the cell permeability (*P* < 0.001) as compared with LPS group (Figure 1(f)). ZO-1 and CLDN4 are the important tight junction proteins, which involve the dynamic and controllable barriers; many factors can affect their structure and function and change the permeability of their paracellular pathways to adapt to the needs of the pathological damage [16]. Our results indicated that the expression level of ZO-1 and CLDN4 significantly decreased by LPS. Meanwhile, this was significantly reversed by XXMD treatment in a dose-dependent manner (*P* < 0.001), compared with LPS group (Figure 1(g)).

### 3.1. XXMD Inhibits Barrier Dysfunction and NLRP3 Inflammasome Activation in HPAEpiC Induced by NLRP3 Overexpression

**Figure 2:**
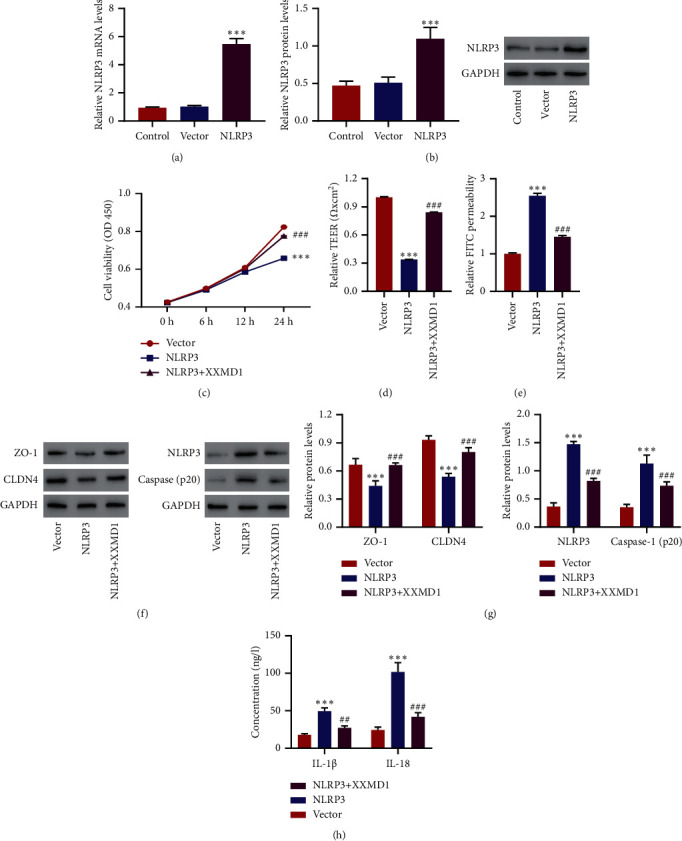
Effects of XXMD on ROS production, hyperpermeability, and NLRP3 inflammasome in HPAEpiC after NLRP3 overexpression. (a, b) HPAEpiC were transduced with pLVX-Puro-NLRP3, and the level of NLRP3 was detected. These cells were transduced with pLVX-Puro-NLRP3 or blank pLVX-Puro vector and then treated with XXMD (1 mg/mL) for 24 h and (c) the cell viability analysis, (d) TEER, (e) FITC permeability, (f, g) the expression level of ZO-1, CLDN4, NLRP3, and caspase 1, and (h) the levels of IL-1*β* and IL-18 were determined. Data are presented as the means ± SD, *n* = 3. ^*∗∗∗*^*P* < 0.001 compared with vector; ^##^*P* < 0.01 and ^###^*P* < 0.001 compared with NLRP3.

In order to assess the effects of XXMD on NLRP3 overexpression-mediated barrier dysfunction and NLRP3 inflammasome activation, HPAEpiC were transduced with pLVX-Puro-NLRP3, and the level of NLRP3 was detected. From the results, it is shown that the level of NLRP3 in HPAEpiC was significantly increased as compared with vector. Western blot demonstrated that the expression level of NLRP3 was markedly enhanced as compared with vector (*P* < 0.001) (Figures 2(a) and 2(b)). In addition, NLRP3 overexpression significantly decreased the cell viability and induced barrier dysfunction, the activation of NLRP3, and the production of IL-1*β* and IL-18, which were inhibited by XXMD (*P* < 0.001) (Figures 2(c)–2(h)).

NLRP3 overexpression inhibits the effect of XXMD on barrier dysfunction and NLRP3 inflammasome in HPAEpiC induced by LPS.

**Figure 3:**
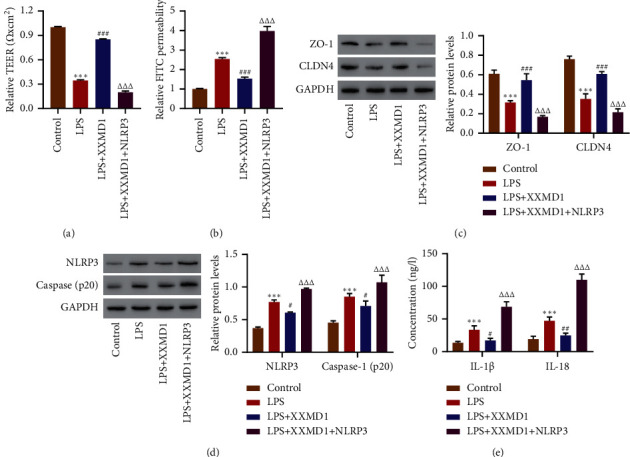
The role of NLRP3 overexpression in the protective effect of XXMD on LPS-induced ROS production, hyperpermeability, and NLRP3 inflammasome. HPAEpiC transduced with pLVX-Puro-NLRP3 or blank vector were treated with XXMD (1 mg/mL) for 3 h and then with LPS (10 mg/L) for 24 h, and the (a) TEER, (b) FITC permeability, (c, d) the expression of ZO-1, CLDN4, NLRP3, caspase 1, and (e) the levels of IL-1*β* and IL-18 were determined. Data are presented as the means ± SD, *n* = 3. ^*∗∗∗*^*P* < 0.001 compared with control; ^#^*P* < 0.05, ^##^*P* < 0.01, and ^###^*P* < 0.001 compared with LPS; ^ΔΔΔ^*P* < 0.001 compared with LPS + XXMD.

To assess the effects of NLRP3 on the protective effect of XXMD in LPS-induced HPAEpiC, HPAEpiC transduced with pLVX-Puro-NLRP3 were treated with XXMD, followed by LPS treatment. Our results demonstrated that XXMD could reduce increase TEER level and decrease cell permeability, the activation of NLRP3, and the level of IL-1*β* and IL-18 as compared with LPS group, however, which was reversed by NLRP3 overexpression (*P* < 0.001) (Figures 3(a)–3(e)). These findings confirmed that XXMD inhibits barrier dysfunction and the activation of NLRP3 induced by LPS.

### 3.2. NAC Decreases the Expression of NLRP3 in HPAEpiC Induced by LPS

**Figure 4:**
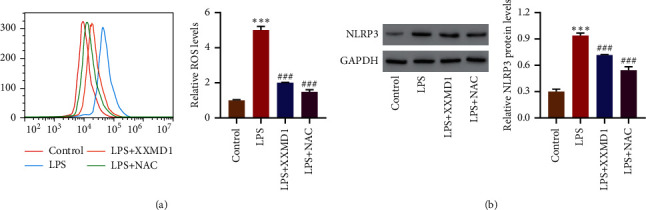
Effects of ROS inhibitor on LPS-induced NLRP3 level. HPAEpiC were treated with XXMD (1 mg/mL) or ROS inhibitor NAC (1 mM) for 3 h and then with LPS (10 mg/L) for 24 h, and the ROS production and NLRP3 expression were determined. Data are presented as the means ± SD, *n* = 3. ^*∗∗∗*^*P* < 0.001 compared with control; ^###^*P* < 0.001 compared with LPS.

In order to assess the effects of ROS on LPS-induced NLRP3, we investigate the effects of NAC on the expression level of NLRP3. From the results, it is shown that XXMD and NAC treatment significantly decrease the ROS levels and NLRP3 expression induced by LPS (*P* < 0.001) (Figures 4(a) and 4(b)). These findings showed that XXMD may suppress the expression level of NLRP3 in LPS-induced HPAEpiC by inhibiting the production of ROS.

### 3.3. XXMD Ameliorated LPS-Induced ALI *In Vivo*

**Figure 5:**
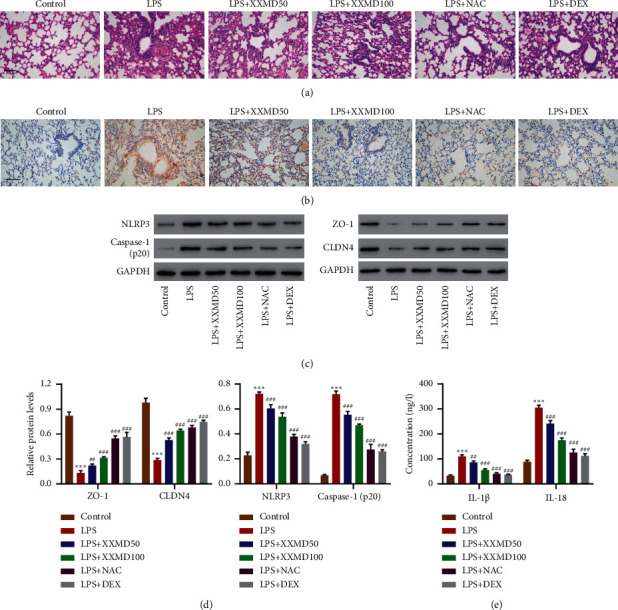
Protective effects of XXMD on LPS-induced acute lung injury *in vivo*. ALI model was induced by LPS intraperitoneal injection (10 mg/kg) with or without intragastric administration of XXMD (50 and 100 mg/kg), NAC (400 mg/kg), or DEX (2.5 mg/kg) 3 days after LPS treatment. (a) H&E, (b) IHC staining for NLRP3, (c, d) the expression levels of ZO-1, CLDN4, NLRP3, caspase 1, and (e) the expression levels of IL-1*β* and IL-18 in bronchoalveolar lavage fluid were detected. Scale bar: 100 *μ*m. Data are presented as the means ± SD, *n* = 6. ^*∗∗∗*^*P* < 0.001 compared with control; ^##^*P* < 0.01 and ^###^*P* < 0.001 compared with LPS.

In order to identify the protective effects of XXMD *in vivo*, ALI model was induced by LPS intraperitoneal injection with or without intragastric administration of XXMD, NAC, or DEX as positive control after LPS treatment. HE was performed to evaluate the lung pathological changes, which showed that there was alveolar septum rupture, severe edema of lung tissue, spot or sheet hemorrhage on the surface of lung tissue, and lots of inflammatory cell infiltration of ALI group as compared with the control group. However, these pathological changes were gradually reduced by XXMD (50 and 100 mg/kg). Meanwhile, NAC as well as DEX also ameliorated these symptoms (Figure 5(a)). Immunohistochemistry staining showed that NLRP3 levels were increased in LPS treatment compared with control group; however, this trend was reversed by XXMD (50 and 100 mg/kg), NAC, and DEX (Figure 5(b)). Western blot showed that there was a higher expression levels of NLRP3 and caspase 1 (p20) and a lower expression levels of ZO-1 and CLDN4 in LPS treatment than the control group; however, this trend was reversed by XXMD (50 and 100 mg/kg), NAC, and DEX (Figures 5(c) and 5(d)). ELISA demonstrated that XXMD (50 and 100 mg/kg) could decrease the level of IL-1*β* and IL-18 induced by LPS, NAC, and DEX which also performed these effects (Figure 5(e)).

## 4. Discussion

ALI is a common clinical disease that causes serious health problems all over the world. It is diffuse damage to the alveolar capillaries caused by multiple factors, and the clinical manifestations are acute dyspnea and refractory hypoxemia [17, 18]. XXMD, as a Chinese medicine, contained multiple activated ingredients, such as liquiritigenin, wogonoside, glycyrrhizic acid, and glyryrrhetinic acid [19]. In a previous study, wogonoside had potentially protective effects on LPS-induced ALI through inhibition of TLR4-mediated NF-*κ*B pathways [20]. Report demonstrated that glycyrrhizic acid exhibited anti-ALI effects by suppressing the inflammatory response [21]. In this study, we aimed to explore whether XXMD could regulate the ROS/NLRP3-mediated NLRP3 inflammasome activation which was associated with LPS-induced ALI. Therefore, we carried out ALI model that was induced by LPS *in vivo*. Meanwhile, HPAEpiC was treated with LPS *in vitro*. Our results showed that XXMD could significantly increase the cell activity against LPS-induced injury *in vitro* and ameliorated lung pathology *in vivo*. The protective effects of XXMD occurred through inhibition barrier dysfunction and NLRP3 inflammasome activation via ROS pathway.

The inflammatory response has a crucial role in LPS-induced lung injury. The activation of NLRP3 is closely related to the occurrence and process of a variety of inflammatory and metabolic diseases [22]. NF-*κ*B signaling pathway is activated by LPS through the pattern recognition receptor of the cell membrane, leading to the expression of inflammasome components, such as NLRP3 and IL-1*β* [23]. In addition, the activation of caspase 1 cleaves pro-IL-1*β* and pro-IL-18 and then formed mature IL-1*β* and IL-18 [24], which were released to the outside of the cell, leading to cell permeability, eventually resulting in cell swelling [25]. Our findings showed that XXMD ameliorated LPS-induced NLRP3 activation and barrier dysfunction though NLRP3. The results of the *in vivo* experiments also confirmed these findings. Since cytoplasmic junction protein directly interacts with the cytoskeleton in the treating ALI [26, 27]. Our results demonstrated that XXMD significantly increase the expression level of ZO-1 and CLDN4, which indicated that XXMD performed protective against ALI through mediating paracellular pathway involved in cell permeability.

ROS, as one of the common NLRP3 inflammasome activators [28, 29], is closely related to the inflammation of lung tissue [30] and generally considered to be an auxiliary messenger that expands inflammation by activating downstream signal cascades, which were involved in the lung injury [31, 32]. Therefore, the ROS/NLRP3 pathway may have an important role in the process of lung injury. Our study demonstrated that XXMD treatment suppressed the production of ROS and expression of NLRP3 and inhibited the level of Caspase-1. Besides, LPS-induced elevation in levels of NLRP3, and proinflammatory cytokines were reversed by treatment with XXMD.

## 5. Conclusion

In conclusion, our study indicated that XXMD ameliorated ALI injury *in vitro* and *in vivo* by suppressing barrier dysfunction and NLRP3 inflammasome activation via ROS/NLRP3 pathway. The results provide the novel insight that XXMD may act as a therapy agent for ALI.

## Data Availability

The data used to support the findings of this study are available from the corresponding author upon request.

## Abbreviations

XXMD: Xiao-Xu-Ming decoction
ALI: Acute lung injury
LPS: Lipopolysaccharide
HPAEpiC: Human pulmonary alveolar epithelial cells
ROS: Reactive oxygen species
ARDS: Acute respiratory distress syndrome
NLRP3: NOD-like receptor family, pyrin domain-containing 3
DMEM: Dulbecco's modified Eagle's medium
CCK-8: Cell counting kit-8
IL-1*β*: Interleukin-1*β*
IL-18: Interleukin-18
ZO-1: Zonula occludens protein 1
CLDN4: Claudin 4
GAPDH: Glyceraldehyde-3-phosphate dehydrogenase
HRP: Horseradish peroxidase
TEER: Transepithelial electrical resistance
FITC: Fluorescein isothiocyanate
ELISA: Enzyme-linked immunosorbent assay
NAC: N-acetyl-L-cysteine
DEX: Dexamethasone
SD: Standard deviation
ANOVA: Analysis of variance.
